# Single-Molecule Fluorescence Imaging Reveals GABAB Receptor Aggregation State Changes

**DOI:** 10.3389/fchem.2021.779940

**Published:** 2022-01-19

**Authors:** Fang Luo, GeGe Qin, Lina Wang, Xiaohong Fang

**Affiliations:** ^1^ CAS Key Laboratory of Molecular Nanostructure and Nanotechnology, Beijing National Research Center for Molecular Science, Institute of Chemistry, Chinese Academy of Science, Beijing, China; ^2^ Department of Chemistry, University of the Chinese Academy of Sciences, Beijing, China

**Keywords:** GABAB receptor, single-molecule imaging, single-molecule tracking, stoichiometry, heterodimer, tetramer

## Abstract

The GABAB receptor is a typical G protein–coupled receptor, and its functional impairment is related to a variety of diseases. While the premise of GABAB receptor activation is the formation of heterodimers, the receptor also forms a tetramer on the cell membrane. Thus, it is important to study the effect of the GABAB receptor aggregation state on its activation and signaling. In this study, we have applied single-molecule photobleaching step counting and single-molecule tracking methods to investigate the formation and change of GABAB dimers and tetramers. A single-molecule stoichiometry assay of the wild-type and mutant receptors revealed the key sites on the interface of ligand-binding domains of the receptor for its dimerization. Moreover, we found that the receptor showed different aggregation behaviors at different conditions. Our results offered new evidence for a better understanding of the molecular basis for GABAB receptor aggregation and activation.

## Introduction

G protein–coupled receptors (GPCRs) are a large family of cell surface signaling proteins which play a central physiological role and represent more than one-third of current drug targets (Cornwell and Feigin, 2020; Wang et al., 2021). Although GPCRs can be generally activated as monomers, recent studies have shown that GPCRs form aggregates to regulate their signaling activity, especially for the class C GPCRs which are characterized by large extracellular ligand-binding domains (LBDs) (Gurevich and Gurevich, 2008; Yong et al., 2013; Lambert and Javitch, 2014; Vischer et al., 2015; Levitz et al., 2016). The metabotropic γ-aminobutyric acid B (GABAB) receptor is a typical C-type GPCR. It is widely expressed in the central and peripheral nervous systems, and its dysfunction is associated with a variety of diseases, such as anxiety, depression, alcohol addiction, Parkinson, and cancer (Hyland and Cryan, 2010; Evenseth et al., 2020; Fritzius and Bettler, 2020; Sanchez-Vives et al., 2021; Shaye et al., 2021). A GABAB receptor consists of two subunits: GABAB_1_ (GBR1) and GABAB_2_ (GBR2). GBR1 is retained in the endoplasmic reticulum after expression. Once it binds with GBR2 to form a heterodimer, the GBR1–GBR2 complex docks to the cell membrane for signaling. Upon ligand stimulation, the GABAB receptor dimer (GBR1–GBR2 complex) recruits G proteins and activates the downstream pathway. Besides, GABAB receptors are also able to assemble into stable higher order oligomers. These oligomers are believed to play an important role in controlling the functional activation of the GABAB receptor (Pin et al., 2004). Therefore, it is of great interest to study the aggregation states and their dynamic changes of GABAB receptors under different conditions.

It has been confirmed by *in vitro* biochemical assays that GABAB receptors form a heterodimer GBR1–GBR2 in the cell (Burmakina et al., 2014). According to the studies of the GABAB receptor crystal structure, each of the receptor subunits, GBR1 and GBR2, consists of three domains: N-terminal LBD, seven helix transmembrane domains (TMDs), and cysteine-rich domain (CRD) connecting LBD and TMD. The heterodimerization of GBR1 and GBR2 for GABAB signaling is mediated by two interaction interfaces in their LB1 and LB2 regions of LBDs (Monnier et al., 2011; Geng et al., 2012; Ellaithy et al., 2020; Kim et al., 2020). However, the dimerization mechanism and the role of dimer interface in receptor function are still unclear. On the other hand, Comps-Agrar et al. (Comps-Agrar et al., 2012) found that GBR1–GBR2 could further form heterotetramers on the cell membrane. Their study was based on the fluorescence resonance energy transfer (FRET) analysis of association and exchange between two populations of the GBR1–GBR2 complex, one is the donor fluorophore–labeled GBR1–GBR2 population presented at the cell surface, and the other is acceptor fluorophore–labeled GBR1-GBR2 population originally retained in the endoplasmic reticulum (ER) but targeted to the plasma membrane after drug application. Several studies demonstrated that the assembly of higher order GABAB receptors resulted in a limited number of signaling protein binding (e.g., one tetramer only binds to 1 G protein), suggesting that the formation of a GABAB tetramer may reduce the receptor activation sensitivity, thus exerting an inhibitory effect (Schwenk et al., 2010; Comps-Agrar et al., 2011; Calebiro et al., 2013). However, the formation of a receptor tetramer has not been observed for other C-class GPCRs. Meanwhile, the aggregation states of GABAB receptors and the A-class GPCR β1 and β2-adrenergic receptors have been reported to be unchanged after ligand stimulation (Calebiro et al., 2013). Therefore, the effect of GABAB dimer aggregation on receptor activation needs to be further studied.

In this study, single-molecule stoichiometry assay is carried out to investigate the formation and change of GABAB dimers and tetramers. Photobleaching event counting and diffusion characterization based on singe-molecule fluorescence imaging is an effective approach to protein stoichiometry determination under physiological conditions (Zhang et al., 2009; Peterson and Harris, 2010; Xia et al., 2013; Hu and Zhang, 2014; Li et al., 2017; Xu et al., 2017; Li et al., 2019; Xu et al., 2019). With the imaging of the GABAB receptor on the cell membrane by using a total internal reflection fluorescence microscope (TIRFM), we revealed the key sites on the hydrophobic interface for GABAB receptor heterodimerization. Furthermore, we found that the receptors can form tetramers in both resting and activated states, and the ligand-induced tetramer population change depends on the receptor expression level and ligand concentration. Our results elucidate the molecular base for GABAB receptor dimerization and its influencing factors, providing new reference for a deeper understanding of GABAB receptor signaling under different conditions.

## Materials and Methods

### Cell Culture

HEK293 cells were cultured in Dulbecco’s modified Eagle medium (DMEM, Gibco) supplemented with 10% fetal bovine serum (Hyclone) and antibiotics (50 mg/ml streptomycin, 50 U/mL penicillin) at 37°C in 5% CO_2_.

### Plasmids, Transfection, and Ligand Treatment

Mutant GBR1 and mutant GBR2 were obtained by mutating Y113 and Y117 of GBR1 and K112, Y118, and D119 of GBR2 into alanine, respectively. The LB2 mutant plasmid was constructed by deleting the sequence encoding the LB2 region in both GBR1 and GBR2 (Geng et al., 2012). Transfection was performed using Lipofectamine 3000 (Invitrogen). In order to obtain low-level GABABR-expressing cells, cells grown in 35-mm glass-bottom culture dish (Shengyou Biotech, China) were transfected with 0.3 μg/ml GBR1-EGFP and GBR2 plasmids in serum-free and phenol red–free DMEM for 6 h. High-level GABABR-expressing cells were transfected with 0.6 μg/ml GBR1-EGFP and GBR2 plasmids for 12 h. After the transfected cells were washed with PBS, the cells were fixed with 4% paraformaldehyde/PBS solution for 30 min or directly used for TIRFM fluorescence imaging in phenol red–free DMEM. For ligand-stimulated cells, the transfected cells were treated with 1 mM gamma-aminobutyric acid (GABA) (Sigma) in phenol red–free DMEM at 37°C for 15 min.

### Single-Molecule Fluorescence Imaging

Single-molecule imaging was performed using a homemade objective-type total internal reflection fluorescence microscope (TIRFM) based on an Olympus IX71 inverted microscope (Zhang et al., 2009; Zhang et al., 2010). The sample was excited by a solid laser (a 488 nm laser), and the emitted light was collected by the oil immersion objective (100x, 1.45na, Olympus) and then separated by the dual-view assembly (Optical Insights). After being filtered by a band-pass filter (525/50 for EGFP), it was projected on to an electron multiplying charge coupled camera (EMCCD, Andor technology du-897 d-bv). The gain of EMCCD was set to 300. The power of excitation light measured after objective lens was modulated to 1 and 2 MW in the epifluorescence mode, respectively, for living cell imaging and fluorescence bleaching step experiments. Andor IQ software and the *z*-axis negative feedback equipment (MFC-2000) were used to control image acquisition. Movies of 300 frames were acquired for each sample at a frame rate of 10 Hz. Fixed cells were also imaged with a confocal microscope (FluoView FV1000-IX81, Olympus, Japan) equipped with a 100x, 1.40NA objective.

### Single-Molecule Tracking and Photobleaching Steps Analysis

Time-lapse image sequences were used to extract the motion information of fluorescent points, and d U-track methods (Jaqaman et al., 2008) were used to track the information. The global optimal tracking algorithm was used to connect the single molecular points between adjacent frames into a complete trajectory. In the first step of the algorithm, the single molecular points between frames were connected; an objective matrix based on lap (linear task allocation) was established; free diffusion or free diffusion plus linear motion was selected according to the motion behavior of the molecules to be tracked; and the points between adjacent frames were connected to segments using the optimal spatiotemporal algorithm. Then, considering the three possible connections of fragments (connection gap, fusion, and separation), the fragments were connected by a global optimal algorithm to form a complete single-molecule tracking trajectory. For the accuracy of fitting, only fluorescence trajectories lasting more than five consecutive frames were collected for a 2D mean square displacement (MSD) calculation. MSD was calculated as follows:

$$\text{MSD}\left(\text{n}\delta \text{t}\right)\;=\frac{1}{N-1-n}\sum_{i=1}^{N-1-n}\left\{{\left[x\left(i\delta t+n\delta t\right)-x\left(i\delta t\right)\right]}^{2}+{\left[y\left(i\delta t+n\delta t\right)-y\left(i\delta t\right)\right]}^{2}\right\}$$

In the formula, Δt (Δt = nδt, with δt = 100 ms) is the elapsed time a single GBR1-EGFP molecule from position x (iδt), y (iδt) moves to x (iδt + nδt), y (iδt + nδt). Here, n and i are integers, and n determines the time increment. N is the total number of image frames before the molecule disappears. According to MSD = 4DΔt, using least squares fitting, the diffusion coefficient D could be calculated based on the slope of the first four points (100 ms). The histogram of the diffusion coefficient was obtained through multiple experiments, and the most possible D-average could be obtained by fitting these histograms with Gaussian distribution. Photobleaching steps of single GABABR molecules on the HEK293 cell membrane were analyzed according to the method we reported previously (Zhang et al., 2009).

### cAMP Analysis

Intracellular cAMP levels were measured using GloSensor cAMP Assay (GloSensor kit, Promega) (Buccioni et al., 2011). The effects of GABA were calculated from signal change relative to the signal obtained with the origin group without GABA.

### Statistics Analysis

Student’s t-tests were performed using GraphPad Prism (GraphPad Software) to compare kinetic data. The nonparametric Mann–Whitney U test was performed using GraphPad Prism (GraphPad Software) to compare the robust data for single-molecule analysis. A *p*-value less than 0.05 was considered statistically significant.

## Results and Discussion

### Single-Molecule Imaging of GABAB Receptor Dimer and Tetramer on Cell Surface

**FIGURE 1:**
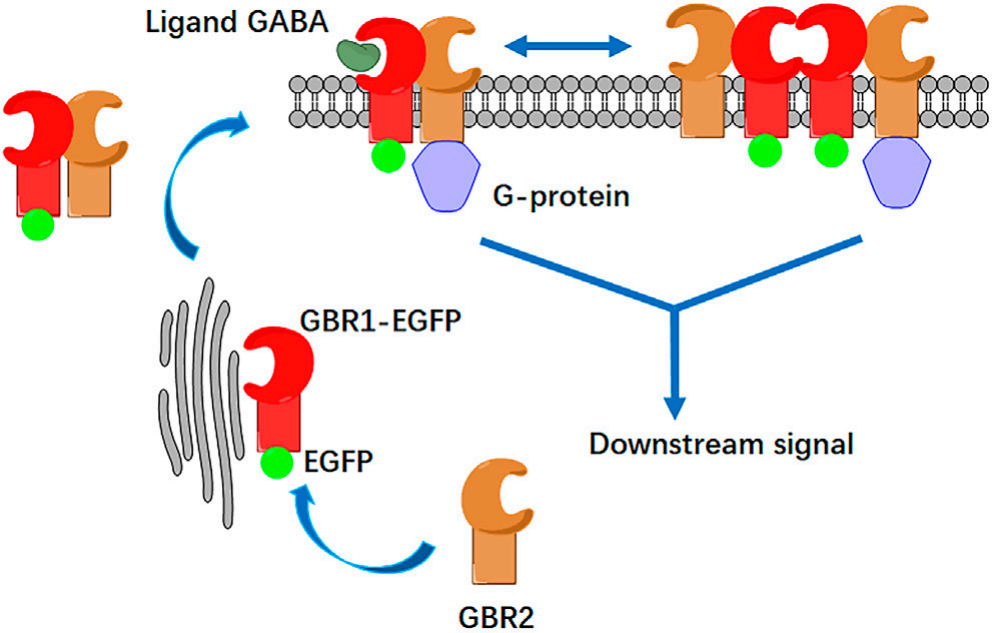
Schematic illustration of GABAB receptor activation and its aggregation at cell membrane. The GABAB receptor consists of two subunits: GBR1 and GBR2. GBR1 carries the endoplasmic reticulum (ER) retention signal (RSRR); thus, it is retained in the endoplasmic reticulum. When GBR1 binds with GBR2 to form a heterodimer, GBR2 blocks the RRSR of GBR1, and both of them could dock to the membrane. On the cell membrane, the heterodimer can also be associated to form a tetramer. After GABA ligand stimulation, heterodimers and tetramers bind to Gi/o proteins to activate the downstream signaling pathway. Either receptor dimer or tetramer only binds with 1 G protein.

**FIGURE 2:**
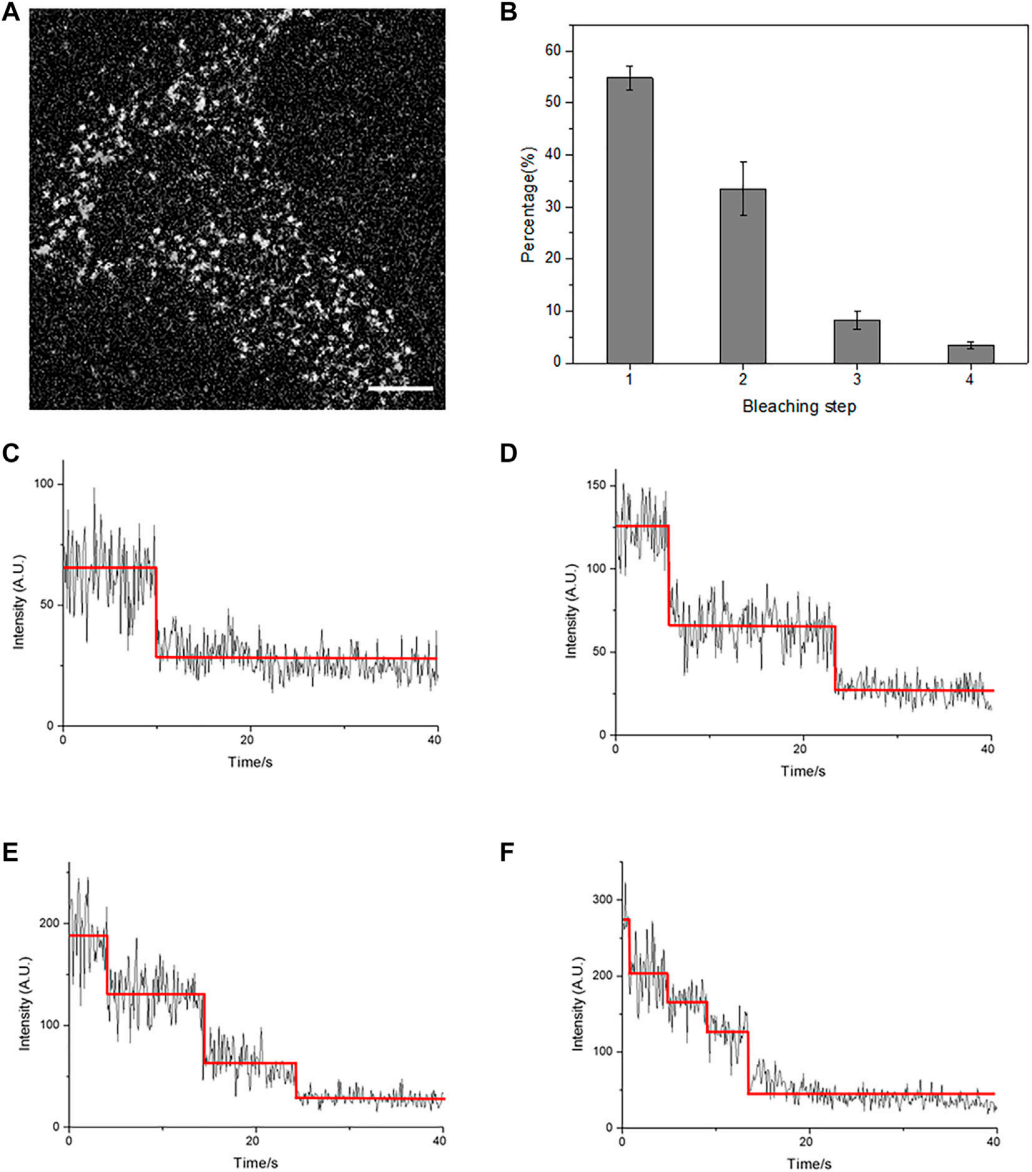
Total internal reflection fluorescence microscopy (TIRFM) imaging revealed a membrane-docked GABAB receptor. **(A)** A typical single-molecule fluorescence image of individual receptor molecules on the HEK293 cell membrane in the absence of GABA. Scale bars: 5 μm. **(B)** The population of bleaching steps of GABAB receptor, GBR1-EGFP/GBR2, on the cell membrane. **(C–F)** Four representative time courses of GBR1-EGFP/GBR2 emission showed one-(C), two-step **(D)**, three-step **(E),** and four-step **(F)** bleaching, respectively. The red lines denote the automatically estimated bleaching steps. The results in **(B)** were calculated from 20 cells with more than 2000 single fluorescent molecules.

According to the activation mode of the GABAB receptor shown in Figure 1, GBR1 and GBR2 could dock on the cell surface only after they form a heterodimer (1:1 ratio) (Pin and Bettler, 2016). Single-molecule fluorescence imaging, based on total internal reflection fluorescence microscopy (TIRFM), was first applied to display single receptor molecules on the cell membrane of HEK293 cells. HER293 cells, which have few endogenous GABAB receptors, are commonly used to study GABABR signaling with the receptor transfection. C-terminal EGFP-labeled GBR1 (GBR1-EGFP) and unlabeled GBR2 were co-expressed in the cells. The results showed that there were a large number of dispersed EGFP fluorescent spots on the cell membrane (Figure 2A). Because GBR1 stayed in the endoplasmic reticulum after expression and it could only dock on the membrane as a heterodimer after binding with GBR2, each single fluorescent spot on the cell membrane would represent a GABAB receptor heterodimer or its aggregates.

Two control experiments were conducted to confirm the fluorescent spots of GBR1-GBR2. When EGFP-labeled GBR1 was expressed alone in HEK293 cells, no fluorescent molecules were observed on the cell membrane by TIRFM. In contrast, the fluorescence signals were found to be concentrated in the cytoplasm by changing TIRFM to epifluorescence mode. This was consistent with the view that the GBR1 would stay in the endoplasmic reticulum (Pin and Bettler, 2016). On the other hand, when GBR2-EGFP was expressed alone at low expression level, dispersed fluorescent spots were observed on the cell membrane, and most of them were bleached in one or two steps. This indicated that GBR2 itself could be docked on the membrane and formed a homodimer. Therefore, GBR2-EGFP is not suitable to co-express with GBR1-EGFP for the judgment of a receptor heterodimer.

Our results proved that the GARBA receptor could be docked on the membrane normally as GBR1-EGFP/GBR2. Next, the individual fluorescent spots on the cell membrane were analyzed by counting fluorescent bleaching steps. Because GBR1-EGFP formed a heterodimer with GBR2 at a ratio of 1:1 when appeared on the cell membrane, the fluorescent spots bleached in one step represented the receptor heterodimer, GBR1-EGFP/GBR2, and the fluorescent spots bleached in two steps represented the tetramer formed by the association of two heterodimers, etc. In the resting state, most of the fluorescent spots were found to show a one-step bleaching (Figure 2C), accounting for about 50% population, while those with two-step bleaching (Figure 2D) accounted for about 30% receptors. These results indicated that the receptor mainly existed in the form of heterodimer on the cell membrane before ligand stimulation, and a considerable part of the receptors formed a tetramer or higher aggregation complex (Figure 2B). Thus, the formation of a heterodimer and tetramer of the GABAB receptor by TIRFM was successfully observed.

### Structural Basis for GABAB Receptor Heterodimer Formation

To investigate the structure basis for receptor dimerization, GBR1-EGFP was truncated by removing LBD or the entire extracellular domain. In both cases, the fluorescence spots on the cell membrane disappeared, indicating that the extracellular domain, especially LBD, was necessary for an effective formation of GBR1-GBR2.

Then the dimer interface between the LBDs of GBR1 and GBR2 was further studied by examining the LBD crystal structure of GABAB receptor (Yong et al., 2013). The GABAB LBD crystal structure showed that it consists of two lobe-shaped domains (LB1 and LB2) which are connected by three short loops. LB1 is located in the upper lobe of LBD, and LB2 is located in the lower one. There are basically two interaction interfaces in LB1 and LB2, respectively, for each receptor. In both resting state and the active state, there are a cluster of conserved hydrophobic residues at the interface in LB1. A total of five residues were chosen to mutate in this cluster, two in the LB1 domain of GBR1 (Y113A and Y117A) and three in the LB1 domain of GBR2 (K112A, Y118A, and D119A), to construct the mutant GBR1M-EGFP and mutant GBR2M-EGFP. In previous studies (Vischer et al., 2015), it has been proved that these sites are related to the function of the receptor, but their effect on receptor dimerization is unknown.

**FIGURE 3:**
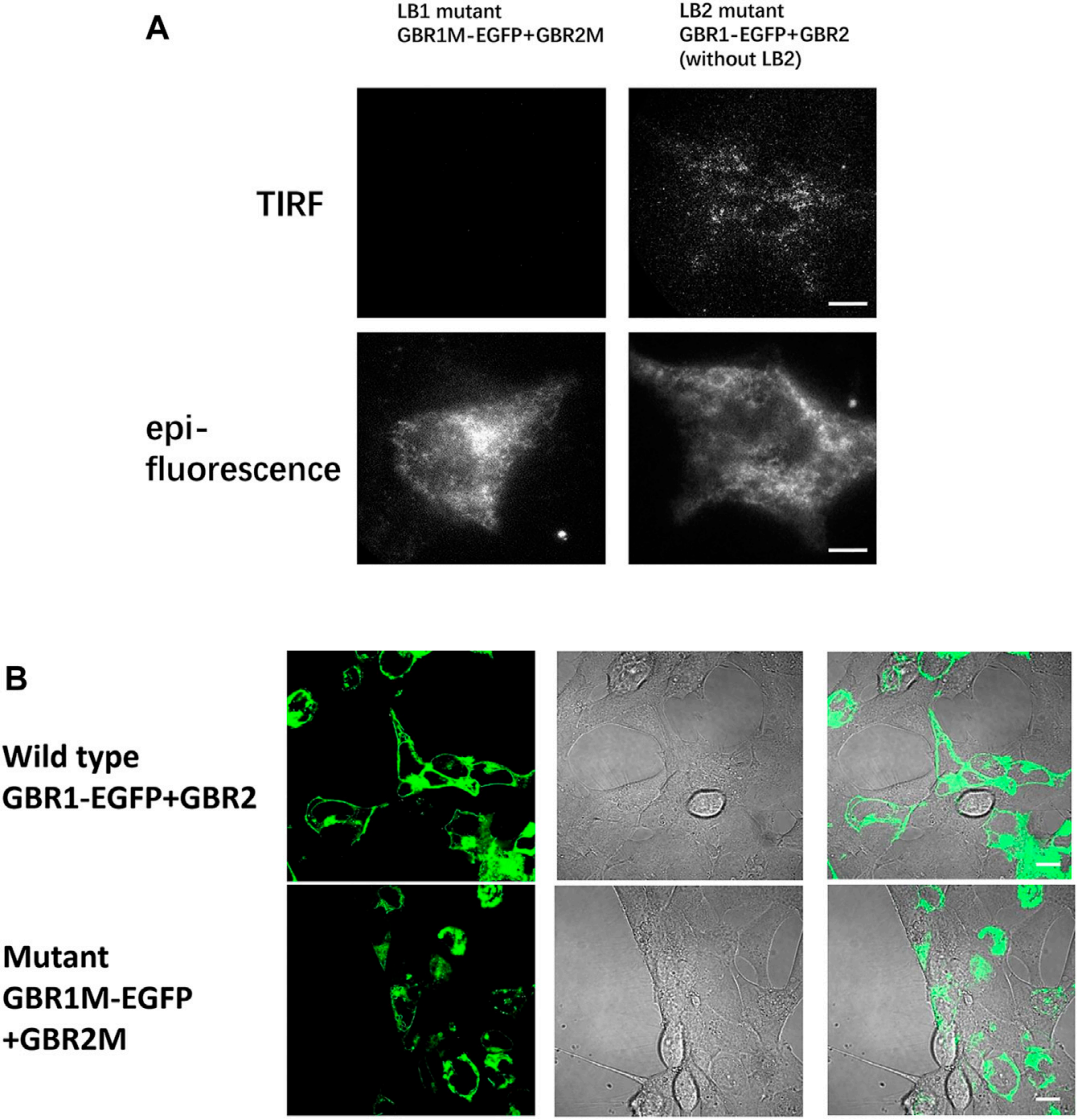
Mutation in the LB1 region effectively prevented receptor dimerization. **(A)** In HEK293 cells, GBR1M-EGFP and GBR2M (with LB1 mutation) were transfected at the same time **(left)**. GBR1M-EGFP remained in the endoplasmic reticulum, and no fluorescent spots were observed on the cell membrane due to the inhibition of dimerization. For the cell transfected with mutant GBR1-EGFP and mutant GBR2 that removed the LB2 region **(right)**, they could still form a heterodimer and dock on the membrane. **(B)** Confocal microscopy was used to observe HEK293 cells transfected with GBR1-EGFP and GBR2 **(upper row)** or mutant GBR1M-EGFP, and GBR2M **(lower row)**. Scale bars: 10 μm.

After single-molecule imaging, the HEK293 cells are co-transfected with either GBR1M-EGFP and wild-type GBR2, or wild-type GBR1-EGFP and GBR2M. The number of fluorescence spots on the cell membrane decreased significantly compared to that shown in Figure 2A. If both mutant GBR1-EGFP and mutant GBR2 were transfected at the same time, almost no fluorescence spots were found in the plasma membrane (Figure 3A), indicating that dimerization was completely inhibited. This is further confirmed by the confocal imaging results (Figure 3B). In the cells that transfected with GBR1-EGFP and GBR2, the fluorescent spots were mainly located on the cell membrane, while in the cells transfected with the mutant plasmids, most of the fluorescent spots remained in the cell interior, indicating that the heterodimerization of the GABAB receptor was inhibited.

**FIGURE 4:**
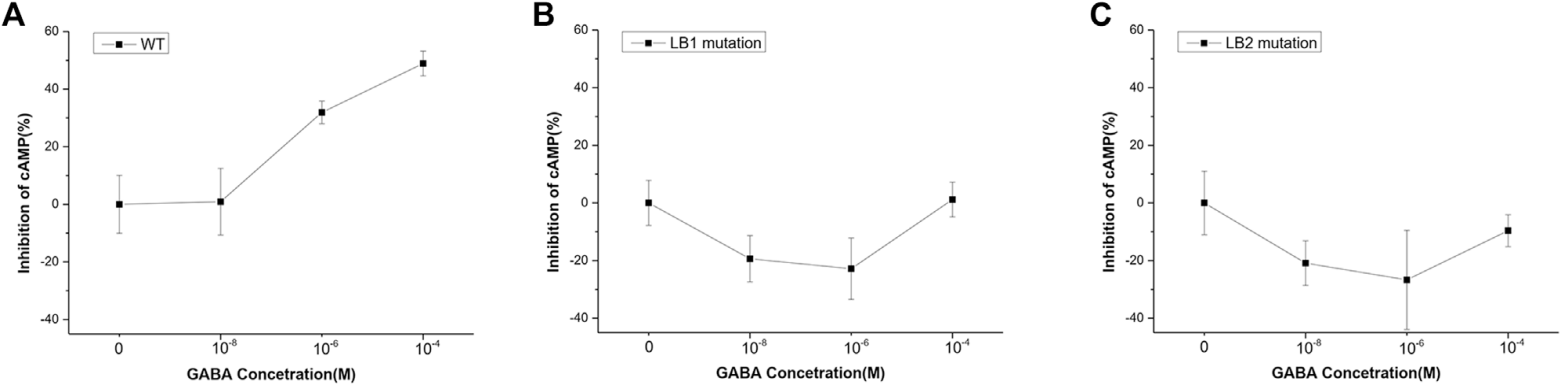
Mutant GBR1M and GBR2M inhibited receptor activation. **(A)** The inhibition effect of HEK293 cells transfected with wild-type GBR1 and GBR2, or **(B)** GBR1M and GBR2M **(B)**, or **(C)** GBR1 nd GBR2 with LB2 mutation under different concentration of GABA. Both mutations in LB1 **(B)** and LB2 **(C)** could seriously affect the function of the receptor. The ligand concentrations used in the experiment were **(from high to low)** 10 ^−4^ , 10 ^−5^ , 10 ^−6^ , 10 ^−7^ , 10 ^−8^ , and 10 ^−9^ M.

The effect of LB1 mutation on GABAB receptor function was tested with the GloSensor kit cAMP assay, which is a biosensor based assay intended to measure cAMP accumulation in cells. Basically, GABABR activation by ligand stimulation inhibited cAMP production and the less cAMP was, the weaker the GloSensor signal we measured, and the percentage of inhibition increases. As shown in Figure 4A,B, the amount of cAMP decreased with the increase of GABA concentration in HEK293 cells transfected with wild-type GBR1 and GBR2, but remained unchanged in the cells which transfected with the mutant GBR1M and GBR2M. It indicated that the GABAB receptor with LB1 mutation has lost the function of activating downstream signal pathway. For the GABAB receptor with LB2 domain truncation (LB2 mutation), although we found its aggregation state was similar to that of wild type, the lower inhibition ratio suggested it did affect the receptor activation and function (Figure 4C).

According to the study on the crystal structure of the GABAR receptor, the interface of GBR1 and GBR2 dimerization is located in the LB1 region at the resting state, and a new interface at LB2 would form after ligand binding (Yong et al., 2013). Our single-molecule imaging data supported that the association of GBR1-GBR2 at the resting state is dependent on LB1 but not on LB2, with the new *in situ* characterization results on the cell membrane. In addition, we found that under the condition of LB2 domain deletion, the GBR1–GBR2 dimer could be still formed (Figure 3A), although GABAR signaling was reduced. Moreover, for the previous key five amino acids at the LB1 interface, previous work reported that each single amino acid mutation led to the reduction of the GABAR signal. Our results revealed that these five sites are the key sites of receptor dimerization, with the mutation of all the five single amino acids, both GBR1–GBR2 dimerization and receptor signaling were fully inhibited.

### Changes of GABAB Receptor Aggregation State After Ligand Stimulation

After the analysis of GABA receptor stoichiometry at the resting state, its aggregation state change after activation was explored. Previous studies showed that the GABAB receptor could be activated by a dimer and tetramer, and no change was found in its aggregation state after ligand stimulation (Calebiro et al., 2013; Pin and Bettler, 2016). In this study, it is interesting to find that the receptor showed different aggregation behaviors at different conditions.

**FIGURE 5:**
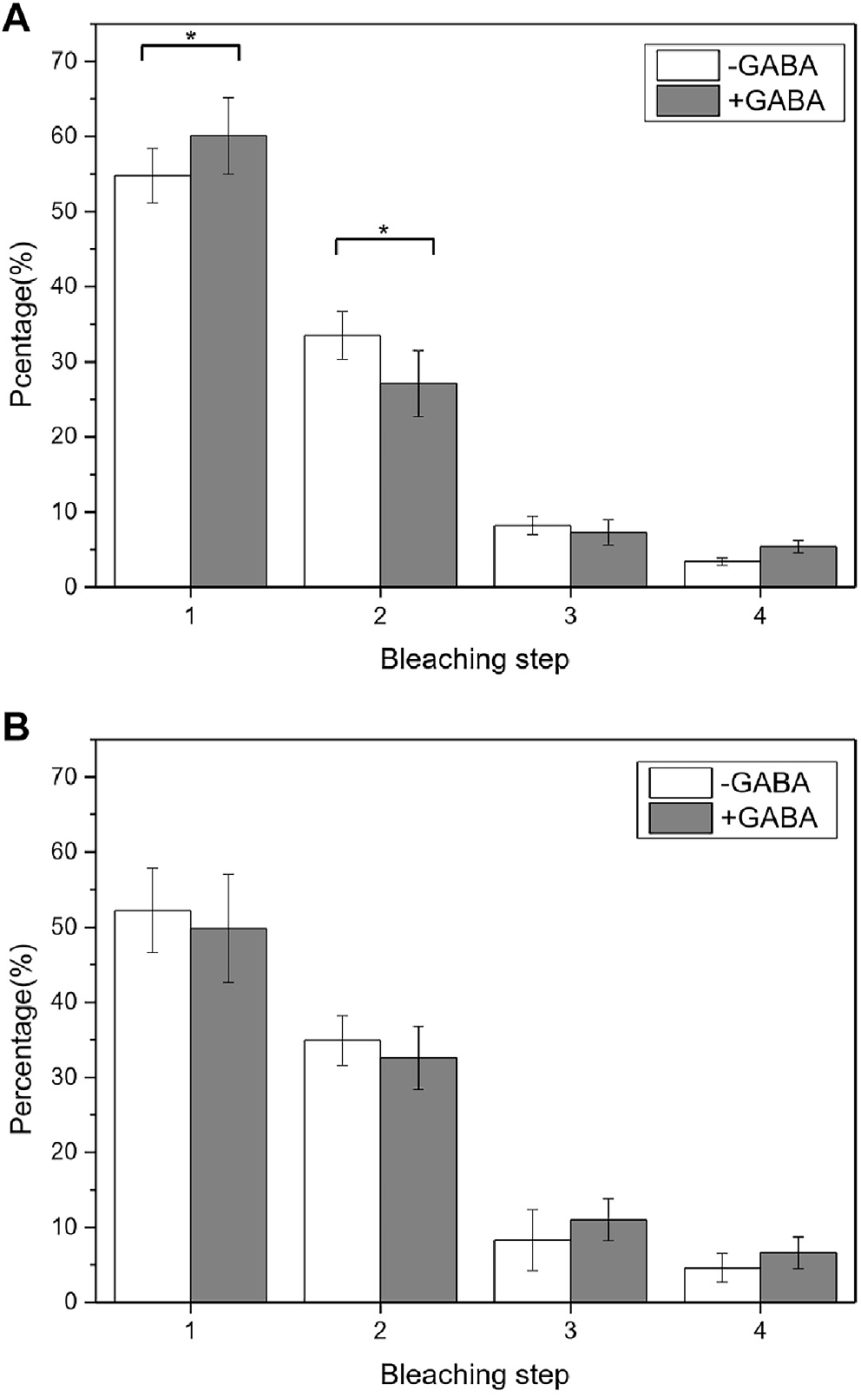
Aggregation state changed before and after the receptor stimulation at different levels of the GABABR expression. **(A)** At the low GABABR expression level, the comparison of GABAB receptor population with different photobleaching steps before and after GABA treatment indicated the aggregation state was changed (*p* = 0.03176 < 0.05, Mann–Whitney U test). **(B)** At the high-level GABABR expression, the comparison of GABAB receptor population with different photobleaching steps. There was no significant change in aggregation before and after ligand stimulation. 20 cells and more than 2000 fluorescent dots were used for the data analysis.

We first checked the GABABR aggregation state change at different expression levels by controlling the receptor transfection time. The transfection time of 6 h resulted in a very low-level GABABR expression, while the transfection time of more than 12 h resulted in a relatively high-level GABABR expression but still suitable for single-molecule imaging. When the expression of EGFP-GBR1/GBR2 was relatively high (the density of the dispersed fluorescent spots was about 0.87 particle/μm^2^), the results from the single-molecule photobleaching counting showed no change in the dimer/tetramer population after 1 mM GABA stimulation (Figure 5B). In contrast, when the expression level was low (0.15 particle/μm^2^), the proportion of one-step bleaching molecule increased and that of two-step bleaching molecule decreased after ligand stimulation (Figure 5A) (*p* < 0.05).

It is known that the downstream pathway can be also activated by the formation of a GABA tetramer, but one heterotetramer can only bind to 1 G protein, so its activation efficiency is lower than that of a heterodimer (Pin and Bettler, 2016). Therefore, we expected that at a low GABABR expression level, GABA stimulation could reduce the number of tetramers and increase the number of dimers on the surface of the cell membrane, which is beneficial to improve the activation efficiency of the receptor. At a high GABABR expression level, when the density of receptors on the cell membrane is high enough to activate downstream pathways, the receptor activation efficiency no longer needs to be enhanced, thus no tetramer-to-dimer change.

### Analysis of Diffusion Changes by Single-Molecule Tracking

**FIGURE 6:**
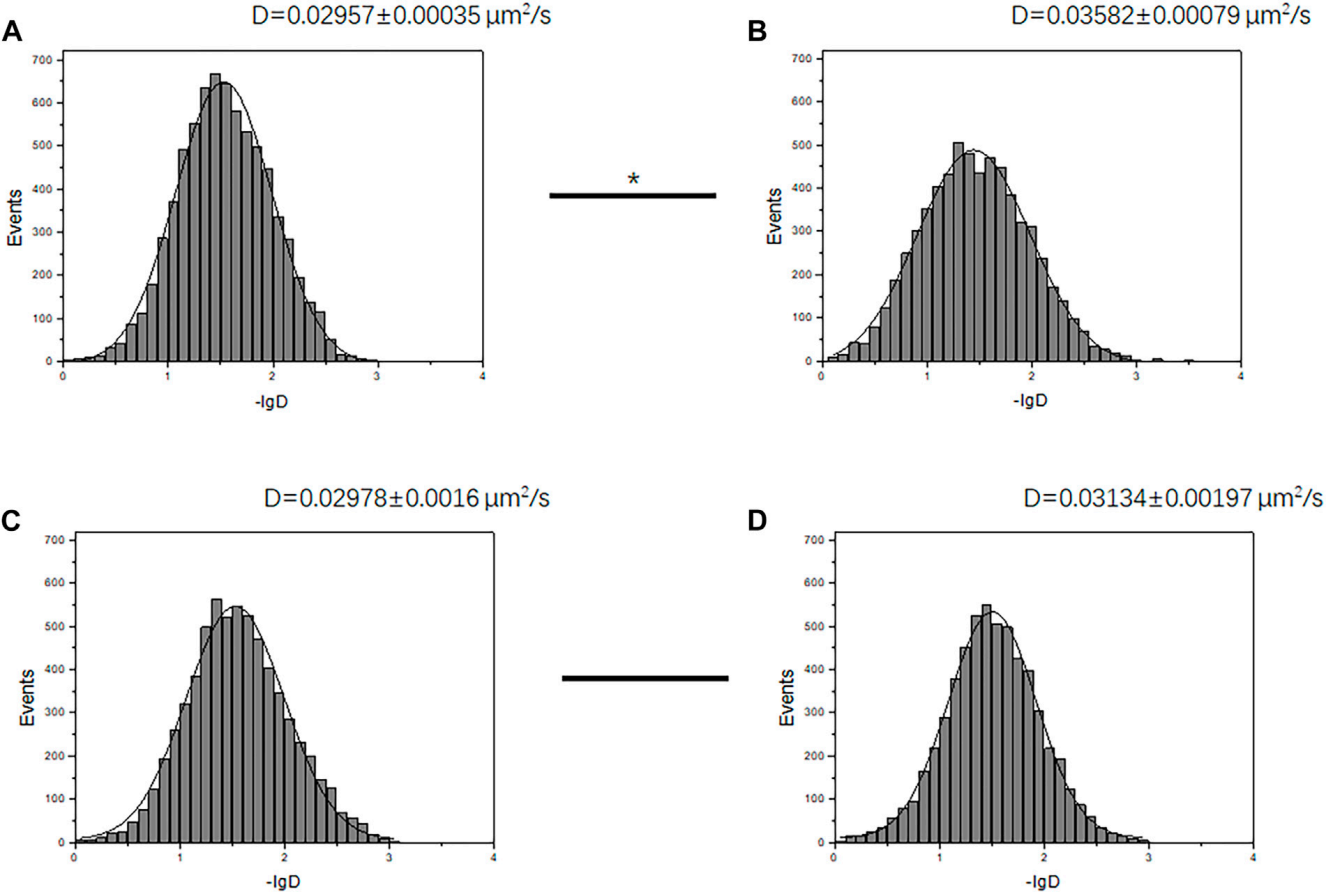
Receptor diffusion rate changed before and after receptor stimulation under different conditions. **(A, B)** Diffusion rates D of membrane-docked GBR1-EGFP/GBR2 molecules in HEK293 cells with the marked D value before **(A)** and after **(B)** GABA stimulation, at a low GABABR expression level (transfection time 6 h). The two groups of D values were significantly different (*p* < 0.05, Mann–Whitney U test). **(C, D)** Diffusion rates D of GBR1-EGFP/GBR2 molecules before **(C)** and after **(D)** GABA stimulation, at a high GABABR expression level (transfection time 12 h). The two groups of D values did not have significant difference. The ligand concentration used in stimulation is 1 mM.

To verify the previous observation on GABAR tetramer and dimer aggregation state change, we further evaluated the diffusion rate of GABABR on the cell membrane with living cells. It is known that the diffusion rate (D) of molecules on the cell membrane surface is affected by molecular volume. With the increase in molecular volume, the diffusion rate will decrease. In the case of low-level GABABR expression, the average diffusion rate of the receptor increased after ligand stimulation (Figures 6A and B) (*D* value changed from 0.02957 ± 0.00035 μm^2^/s to 0.03582 ± 0.00079 μm^2^/s, *p* < 0.05, *t*-test). This is consistent with the change in the aggregation state we found in Figure 5. Since tetramers and higher order oligomers are not beneficial to the activation of receptors, GABABRs turn from tetramers to more efficient dimers after ligand stimulation, resulting in a faster diffusion rate due to the reduction in volume. In the case of high-level GABABR expression, GABA stimulation did not change the movement state of most receptors (*D* value changed from 0.02978 ± 0.0016 μm^2^/s to 0.03134 ± 0.00197 μm^2^/s, *p* > 0.05, *t*-test) due to the excessive number of receptor dimers on the membrane (Figure 6 C, and D) and no tetramer–dimer population change was observed.

In conclusion, we have used the single-molecule imaging method to reveal the molecule basis for GABAB receptor dimerization and aggregation state change. Moreover, we found that ligand stimulation can result in the change of receptor tetramer into dimer at a low receptor expression level, in order to improve the activation efficiency. Our results provide new reference for the understanding of the regulation of GABAB receptor aggregation and activation.

## Data Availability

The original contributions presented in the study are included in the article/Supplementary Material, and further inquiries can be directed to the corresponding author.
